# Combined sequential use of HAP and ART scores to predict survival outcome and treatment failure following chemoembolization in hepatocellular carcinoma: a multi-center comparative study

**DOI:** 10.18632/oncotarget.9604

**Published:** 2016-05-26

**Authors:** David J. Pinato, Tadaaki Arizumi, Jeong Won Jang, Elias Allara, Puvan I. Suppiah, Carlo Smirne, Paul Tait, Madhava Pai, Glenda Grossi, Young Woon Kim, Mario Pirisi, Masatoshi Kudo, Rohini Sharma

**Affiliations:** ^1^ Department of Surgery and Cancer, Imperial College London, Hammersmith Hospital, London, UK; ^2^ Department of Gastroenterology and Hepatology, Kinki University School of Medicine, Osaka-Sayama, Osaka, Japan; ^3^ Department of Internal Medicine, The Catholic University of Korea Incheon St. Mary's Hospital, Seoul, Republic of Korea; ^4^ Department of Translational Medicine, Università degli Studi del Piemonte Orientale “A. Avogadro”, Novara, Italy; ^5^ School of Public Health, Università degli Studi di Torino, Torino, Italy; ^6^ Department of Radiology, Imperial College NHS Trust, Hammersmith Hospital, London, UK; ^7^ Department of Surgery, Imperial College NHS Trust, Hammersmith Hospital, London, UK; ^8^ Interdisciplinary Research Center of Autoimmune Diseases, Università degli Studi del Piemonte Orientale “A. Avogadro”, Novara, Italy

**Keywords:** prognosis, hepatocellular carcinoma, TACE, ART score, HAP score

## Abstract

**Background:**

The prognosis of patients with hepatocellular carcinoma (HCC) undergoing transarterial chemoembolization (TACE) is variable, despite a myriad of prognostic markers. We compared and integrated the established prognostic models, HAP and ART scores, for their accuracy of overall survival (OS) prediction.

**Results:**

In both training and validation sets, HAP and ART scores emerged as independent predictors of OS (*p<0.01*) with HAP achieving better prognostic accuracy (c-index: 0.68) over ART (0.57). We tested both scores in combination to evaluate their combined ability to predict OS. Subgroup analysis of BCLC-C patients revealed favorable HAP stage (*p<0.001*) and radiological response after initial TACE (*p<0.001*) as positive prognostic factors.

**Patients and Methods:**

Prognostic scores were studied using multivariable Cox regression and c-index analysis in 83 subjects with Barcelona Clinic Liver Cancer (BCLC) A/B stage from UK and Italy (training set), and 660 from Korea and Japan (validation set), all treated with conventional TACE. Scores were further validated in an separate analysis of patients with BCLC-C stage disease (n=63) receiving initial TACE.

**Conclusion:**

ART and HAP scores are validated indices in patients with intermediate stage HCC undergoing TACE. The HAP score is best suited for screening patients prior to initial TACE, whilst sequential ART assessment improves early detection of chemoembolization failure. BCLC-C patients with low HAP stage may be a subgroup where TACE should be explored in clinical studies.

## INTRODUCTION

Patients presenting with liver-confined hepatocellular carcinoma (HCC), preserved liver function and performance status cluster into “intermediate stage” or Barcelona Clinic Liver Cancer (BCLC) B category [1]. In this patient subgroup where overall survival (OS) often extends beyond 2 years [2], guidelines recommend trans-arterial chemoembolization (TACE) with the intent of prolonging OS by achieving local disease control [3].

TACE is also indicated in early HCC when surgery or radiofrequency ablation is contraindicated [4]. In parallel, whilst not supported by randomized controlled trials (RCT), a growing number of single-center studies indicate that embolization is safe and effective also in patients with segmental portal vein invasion or systemic metastases [5]. Because of the evolving experience in the administration of TACE, achieving consensus regarding optimal selection criteria and best re-treatment strategy is critical in improving and maintaining clinical outcomes [6].

However, due to the heterogeneity in the prognosis of BCLC-B HCC, where predicted OS ranges from 11 to 45 months, treatment decisions in the individual patient are partially subjective, being influenced by the local expertise within each institution [7]. Previous studies have identified a number of individual adverse prognostic traits in patients undergoing TACE [8]. These have been variously combined to derive coherent prognostic models aiming to standardize the prognostic assessment across institutions [7, 9–12]. Based on these scores, subjects with shorter survival expectancy might be offered systemic treatment or best supportive care, avoiding exposure to the adverse effects of TACE.

Of the prognostic scores, the hepatoma arterial-embolization prognostic (HAP) score is a model constructed on baseline pre-TACE hypoalbuminemia <35 g/L, bilirubin >17 mmol/L, AFP >400 ng/ml and tumor size >7 cm designed to guide initial TACE treatment [11]. A second, recently qualified prognostic model, the ART score, is based on the deterioration of liver biochemistry following initial TACE and the presence of radiological response to treatment [12], and can be used to identify patients who may benefit from sequential retreatment [13] (Table 1).

**Table 1:** The hepatoma arterial-embolisation prognostic (HAP) score and the assessment for re-treatment with TACE (ART) score

Prognostic Model	Variables	Prognostic Stratification
HAP Score	Albumin <35 g/L AFP >400 ng/dL Tumor diameter >7cm Bilirubin >17mmol/L	HAP A HAP B HAP C HAP D
ART Score	Child Pugh increase following TACE (+1, +2 points) AST >25% from baseline Lack of radiologic response	High risk (>2.5) Low risk (<2.5)

Whilst scientifically interesting, the relationship between these scores and patient's overall survival has been questioned following validation in independent cohorts, casting doubt upon their clinical utility [14, 15]. As a result, the use of either score is not advocated within the current management guidelines for HCC. In addition, there are no available data to suggest which score is best in predicting patient prognosis, or how best to combine both scores in the clinical setting.

We designed this multi-institutional study aiming to validate the accuracy of HAP and ART score in predicting patients' survival after initial TACE. We employed independent cohorts of unselected, consecutive patients with HCC presenting within intermediate stage criteria from Europe and Asia to ensure ample generalizability of the results, evaluating both scores individually and in combination. Secondarily, we intended to investigate whether the proposed scores preserved prognostic prediction in a subgroup of patients exceeding BCLC-B criteria treated with TACE in a post-hoc analysis.

## RESULTS

### Demographics

The clinicopathologic features of both datasets are illustrated in Table 2. In the training set, median age was 72 years, 50% of the patients were staged as intermediate stage HCC according the BCLC algorithm, with preserved liver function (CTP A, 75%). Minimum follow-up time was 4 months or until date of death. At the time of analysis 37% of patients had died. Median OS was 26 months (4-162 months).

**Table 2:** Demographic and clinical characteristics of patients with HCC treated with TACE (training and validation set)

Baseline characteristic	n=83, (%) or median, (range)	n=660, (%) or median, (range)
**Age,** years	72 (47-84)	73 (42-89)
**Gender**		
Male	64 (77)	465 (70)
Female	19 (23)	195 (30)
**Aetiology of Chronic Liver Disease**		
Viral	49 (60)	
Non Viral	31 (37)	533 (80)
Not characterized	3 (3)	127 (20)
**Child Turcotte Pugh Class**		
A5	37 (45)	332 (49)
A6	25 (30)	168 (26)
B7	14 (17)	94 (14)
B8	5 (6)	43 (7)
B9	2 (2)	23 (4)
**Maximum tumour diameter**		
≤ 5 cm	26 (40)	565 (86)
> 5 cm	38 (60)	95 (14)
**Number of nodules**		
1	28 (34)	110 (17)
2	22 (26)	120 (18)
3	16 (20)	97 (15)
>3	17 (20)	248 (38)
Missing	−	85 (12)
**AFP**		
<400 ng/mL	77 (93)	534 (81)
≥400 ng/mL	6 (7)	126 (19)
**Albumin**, g/L	37 (23-49)	37 (20-50)
**Total bilirubin,** umol/L	19 (7-55)	14 (3-70)
**ALT**, IU/L	48 (13-177)	37 (4-277)
**AST**, IU/L	56 (16-188)	48 (6-303)
**ALP**, IU/L	255 (113-529)	336 (108-1212)
**INR**	1.2 (1.0-1.6)	1.0 (1.0-2.0)
**Platelet Count**, x 10^9^/L	115 (26-269)	115 (14-453)
**BCLC Stage**		
A	42 (50)	270 (40)
B	41 (50)	390 (60)
**CLIP Score**		
0-1	61 (73)	N.A.
≥2	22 (27)	
**Number of TACE procedures**		
1	42 (50)	171 (26)
2	16 (20)	139 (21)
≥3	25 (30)	274 (42)
Missing	−	76 (11)
**Prior Treatments**		
First line TACE	50 (60)	322 (48)
Resection	6 (7)	77 (12)
Transplantation	1 (1)	0 (0)
Radiofrequency ablation	22 (27)	243 (36)
Systemic treatment	4 (3)	18 (4)
**Modified RECIST response following TACE**		
Complete Response	22 (26)	268 (40)
Partial Response	40 (48)	110 (17)
Stable Disease	12 (15)	91 (14)
Progressive Disease	9 (11)	191 (29)
**HAP Score**		
A	23 (28)	274 (41)
B	32 (39)	209 (32)
C	24 (30)	137 (21)
D	3 (3)	40 (6)
**ART Score**		
<2.5	55 (66)	423 (64)
>2.5	28 (44)	237 (36)

### Survival analysis

**Figure 1:**
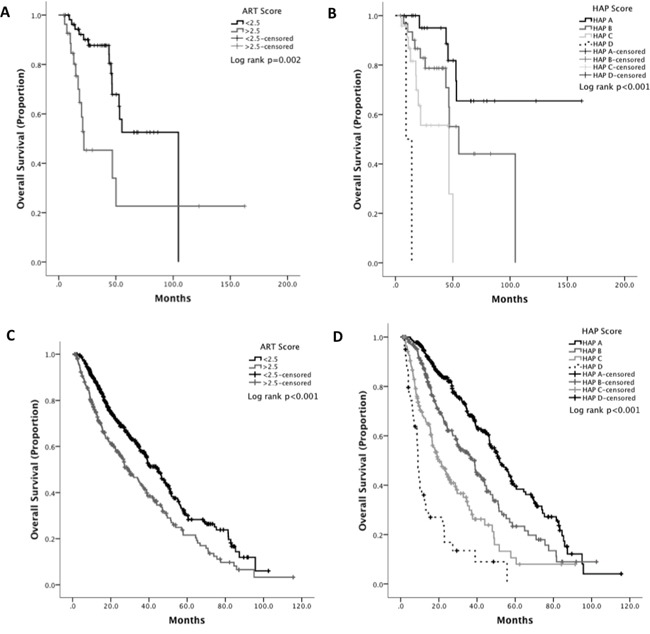
Kaplan Meier curve analysis showing the effect of ART and HAP score as predictors of overall survival in HCC in the training **(A, B)** and in the validation set **(C, D)**.

On univariable analyses, intrahepatic spread (*p<0.007*), tumor size >5cm (*p<0.001*), AFP>400 ng/ml (*p<0.001*), CTP class (*p=0.008*), radiologic response post-TACE (*p=0.002*), CLIP (*p<0.001*), HAP (*p<0.001*) and ART score (*p=0.002*) emerged as significant predictors of OS in the training set (Table 3). Based on ART score, the median OS for patients of good prognosis (ART score <2.5) was 55 months, reducing to 22 months in patients with a poor score (Figure 1A). In patients with HAP stage A disease, median OS was not reached at the end of observation. Patients within HAP stage B or C had a median OS of 55 and 46 months respectively, deteriorating to 9 months in stage D disease (Figure 1B).

**Table 3:** Univariate analysis of prognostic factors of overall survival (training set)

	UNIVARIATE ANALYSIS			
Variable	N=83	Median OS (95% CI)	Hazard Ratio (95% CI)	P-value
**Age**				
<65	32	−	−	0.45
>65	51			
**Etiology**				
Viral	31	−	−	0.91
Non-viral	49			
**Intrahepatic spread**				
Uninodular <50%	27	104 (12-196)	2.6 (1.3 – 5.2)	0.007*
Multinodular <50%	51	50 (40-59)		
Massive ≥50%	8	19 (5-35)		
**Maximum tumour diameter**				
<5 cm	67	55 (20-90)	3.9 (1.7-8.7)	<0.001*
≥5 cm	20	21 (16-27)		
**AFP,** ng/ml				
<400	78	55 (17-93)	8.2 (3.3 – 20.8)	<0.001*
≥400	8	14 (7-21)		
CLIP Score*				
0-1	61	104 (32-176)	2.3 (1.5-2.5)	0.001*
≥2	22	22 (18-25)		
**BCLC**				
A	42	−	−	0.22
B	41			
**Child Turcotte Pugh Class**				
A	62	104 (33-175)	2.9 (1.3-6.5)	0.008*
B	21	46 (10-83)		
**Tumour Response (mRECIST)**				
CR	22	NR	1.9 (1.2-2.9)	0.002*
PR	40	55 (36-73)		
SD	12	52 (26-79)		
PD	9	21 (7-37)		
**HAP Score**				
A	23	NR	3.1 (1.8-5.3)	<0.001*
B	32	55 (34-75)		
C	24	46 (18-82)		
D	3	9 (43-63)		
**ART Score**				
<2.5	55	104 (30-160)	3.1 (1.5-6.7)	0.002*
>2.5	28	22 (10-47)		

NR: Not reached.

A multivariable regression model was constructed including HAP, ART score, mRECIST and CLIP score (categorized as 0-1 versus ≥2) with other significant univariable predictors being excluded from analysis to avoid collinearity. Cox regression analysis confirmed HAP and ART scores (Supplementary Table 1) together with mRECIST (Hazard Ratio [HR] 1.5 95%CI 1.0-2.3, *p=0.04*) as independent predictors of patients' OS.

### Validation of prognostic models

The prognostic accuracy of the ART and HAP scores was further tested in a larger, independent validation dataset. As shown in Table 2, there was homogeneity across both datasets in terms of age, gender distribution, liver functional reserve (CTP A, 75%), stage (BCLC-B, 60%) and median OS which in the validation set, 23 months (range 1-115 months).

Both ART and HAP score remained significant on univariate (*p<0.001*) and multivariate (*p<0.001*) analysis of OS with adjusted hazard ratios shown in Supplementary Table 2. Patients with an ART score >2.5 after initial TACE had an OS of 29 months (range 23-34) compared to those with an ART score <2.5 whose median OS was 45 months (range 40-50, *p<0.001*) (Figure 1C). Patients within HAP stage A or B had median OS of 52 (95%CI 45-58) and 37 (30-44) months compared with stage C or D with median OS of 19 (14-25) and 9 (8-10) months, respectively (p<0.001) (Figure 1D). Radiologic response to treatment emerged as additional multivariable predictor of OS (HR 1.7 95%CI 1.6-1.8 *p<0.001*).

We assessed the accuracy of the HAP and ART score in predicting early mortality using ROC curve analysis based on 1 and 2-year survival rates. In the training set, both scores had acceptable accuracy in estimating mortality after initial TACE, with AUROC values of 0.67 (95%CI 0.50-0-85) for the ART score and 0.75 (95%CI 0.62-0.89) for the HAP score at 1 year, and 0.75 (95%CI 0.63-0.86) for the ART score and 0.73 (95%CI 0.62-0-85) for the HAP score at 2 years (*p<0.05*) (Supplementary Figure 1A-B). In the validation set, the HAP score was a more accurate predictor of 1-year mortality with AUROC values of 0.70 (95%CI 0.66-0.75) compared to the ART score 0.57 (95%CI 0.52-0.62, *p<0.05*). Similarly, 2-year mortality was more accurately predicted by the HAP score (0.67, 95%CI 0.63-0.71) than the ART score (0.53, 95%CI 0.50-0.58, *p<0.05*) (Supplementary Figure 1C-D).

The discriminatory capacity of each prognostic system was compared by means of Harrell's concordance index [22]. The HAP score displayed an overall better discriminatory ability in predicting OS with a c-score of 0.68 (95%CI 0.64-0.70) compared to the ART score (c-score 0.57, 95%CI 0.53-0.60). The combination of HAP and ART score yielded a pooled c-score of 0.69 (95% CI 0.65-0.72).

We further confirmed the prognostic validity of both HAP and ART score in a pooled analysis of patients belonging to both training and validation sets (n=746). As shown in Supplementary Figure 2, this confirmed the prognostic value of both scores in the entire study population (Log rank p<0.001 for both HAP and ART scores). Given the independent prognostic role observed for the HAP and ART scores we evaluated the accuracy of their combined sequential use in the entire study population. As shown in Supplementary Table 3, the ART score was able to detect a significant survival difference of 15 months in HAP B (p<0.001), 17.1 months in HAP C (p=0.001) and 1 month in HAP D (p=0.02) patients, with no effect on survival seen for patients clustering in HAP A stage (p=0.96) (Supplementary Figure 3).

### Evaluation of prognostic models in patients exceeding *BCLC-B* criteria

**Figure 2:**
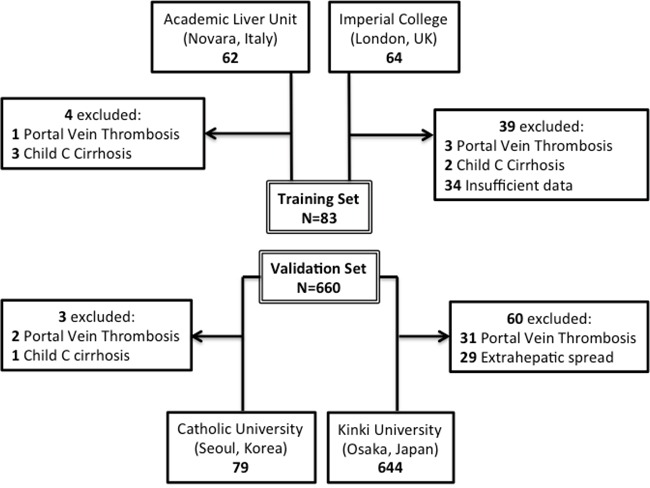
Study flow diagram illustrating patient inclusion in the training and validation set

We performed a separate survival analysis on 63 patients derived from the validation set who had been offered TACE despite exceeding BCLC-B staging criteria due to visceral metastatic spread (*n=29,* 46%), segmental portal vein involvement (*n=34*, 54%) or Child C cirrhosis (*n=1*, 1%). These patients had been excluded from the principal analysis (Figure 2). The median survival of this patient subgroup was 11.8 months (range 1.4-59 months) with a total of 45 deaths (71%) at the time of data analysis. Full clinicopathologic characterization is provided in Table 4.

**Table 4:** Clinicopathologic characteristics of patients exceeding BCLC-B criteria

Baseline characteristic	n=63, (%) or median, (range)
**Age,** years	53 (34-87)
**Gender**	
Male	48 (76)
Female	15 (24)
**Aetiology of Chronic Liver Disease**	
Viral	48 (76)
Non Viral	15 (24)
**Child Turcotte Pugh Class**	
A	51 (80)
B	11 (19)
C	1 (1)
**Maximum tumour diameter**	
≤ 5 cm	33 (52)
> 5 cm	30 (48)
**Number of nodules**	
1	5 (8)
2	15 (24)
≥3	43 (68)
**Extrahepatic Metastasis**	
Absent	34 (54)
Present	29 (46)
**Portal Vein Involvement (segmental)**	
Absent	29 (46)
Present	34 (54)
**AFP**	
<400 ng/mL	43 (68)
≥400 ng/mL	20 (32)
**Albumin**, g/L	37 (21-46)
**Total bilirubin**, umol/L	13 (3-53)
**ALT**, IU/L	44 (10-122)
**AST**, IU/L	56 (14-138)
**ALP**, IU/L	220 (120-1901)
**INR**	1.1 (1.0-1.6)
**Platelet Count, x** 10^9^/L	121 (14-1653)
**Number of TACE procedures**	
1	31 (50)
2	17 (20)
≥3	15 (30)
**Modified RECIST response following TACE**	
Complete Response	3 (5)
Partial Response	21 (32)
Stable Disease	11 (18)
Progressive Disease	28 (45)
**HAP Score**	
A	12 (19)
B	20 (32)
C	22 (35)
D	9 (14)
**ART Score**	
<2.5	32 (51)
>2.5	31 (49)

**Figure 3:**
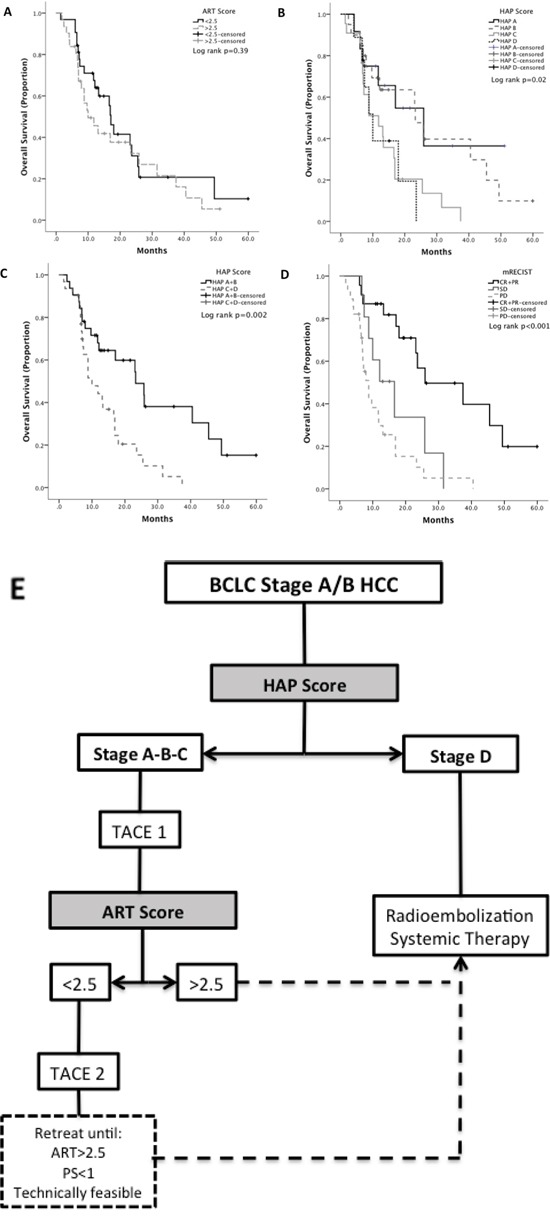
Kaplan Meier curve analysis showing the effect of different prognostic models on the overall survival of a subgroup of patients exceeding intermediate HCC staging criteria: ART score **(A)**, HAP score **(B, C)** mRECIST based radiologic response **(D)**. Panel **E** illustrates a proposed algorithm for the sequential use of the HAP and ART score in BCLC-A/B stage patients.

Survival analysis revealed that response to TACE (HR 2.0 1.4-2.8, *p<0.001*) and the HAP (*p=0.02*) but not the ART score (*p=0.39*) predict OS after initial TACE. Median OS was 25.9 (range 8.6-43.1) in HAP A stage, 23.3 months (range 19.0-27.6) in HAP stage B, 11.8 months (5.4-11.8) in stage C and 10 months (6.5-13.4) in stage D (HR 1.5, 95%CI 1.1-2.2, *p=0.02*). However, in patients with BCLC-C, OS was not significantly different across HAP stages A and B (*p=0.75*), nor across C and D (*p=0.06*). The accuracy of OS estimation was optimized by dichotomizing the HAP score into stages A+B (median OS 23 months, range 12-34 months) against C+D (median OS 10 months, range 5-14 months, HR 2.6 95%CI 1.4-4.9 *p=0.002*). According to mRECIST criteria, median OS was 8 months (range 6-11 months) in patients with progressive disease, 16 months (range 8-25 months) with stable disease and 26 months with complete and partial response (range 6-45 months, *p<0.001*)(Figure 3).

## DISCUSSION

The use of TACE in unresectable HCC as a palliative measure to improve survival whilst maintaining quality of life is supported by level I evidence from 2 primary RCTs and 3 meta-analyses [3]. However, significant heterogeneity in median OS has been reported in patients receiving TACE, varying from 16-40 months in early stage disease to 15-27 months in intermediate [23] and 4-15 months in advanced disease [5]. The wide range in OS figures observed within each stage stems from inter-institution variability in patient selection, diverse re-treatment criteria, and the evolving technique in delivering TACE.

Given the palliative intent of TACE it is important that patient selection is not only driven by the technical feasibility of the procedure, but also guided by careful consideration of the potential survival benefit against the risk of post-procedural adverse events. For this reason, the development of biomarkers to allow for a more objective selection of TACE candidates based on the likelihood to benefit from treatment has been at the focus of intense research efforts.

In our multi-institutional retrospective study of prognostic factors, the largest to our knowledge, we validated for the first time the significance of two recently qualified models, the HAP and the ART score in both Western and Asian patient populations, where survival outcomes can significantly differ as a result of the diverse disease etiology, screening policy and subsequent clinical management [24]. These scores have been proposed as objective and mutually exclusive strategies to select patients for initial as well as subsequent loco-regional therapies based on their capacity to predict long-term survival following TACE. Their prognostic potential, however, has never been compared, a point of major consequence given the emergence of discrepancies from their independent evaluation in separate studies, which has lead to uncertainties in terms of which strategy should be prioritized for clinical use [14, 15].

Whilst meant to inform treatment decisions in intermediate stage HCC, the original study that qualified the HAP score included only 31% of the patients with BCLC-B stage, with lack of complete staging data in the validation set. In addition, 36% of the patients exceeded BCLC-B criteria, though variables including portal vein involvement, PS or extra-hepatic disease were not documented in the analysis, potentially causing a greater heterogeneity in survival [11].

Similar pitfalls may influence the validity of the ART score, where recent retrospective studies have in fact demonstrated that only 10-15% of the patients were eligible for ART score testing [14, 15] based on the original eligibility criteria, which excluded patients <2 TACE within 90 days [12], suggesting a reduced applicability of the model in the general population.

Unlike other studies, we included TACE candidates consecutively and in an unselected manner apart from BCLC stage, therefore allowing for the prognostic scores to predict for outcome after initial TACE more closely to a “real life” clinical practice scenario. In our study, both scores emerged as highly significant predictors of OS (Figure 1), preserving an independent effect when tested on a multivariable Cox regression model (Supplementary Tables 1, 2). Our data show that the HAP score displayed better accuracy in estimating short-term mortality at 1 and 2 years following TACE (Supplementary Figure 1), a finding that was confirmed by c-index analysis indicating a superior predictive accuracy of the HAP over the ART score in estimating long term OS.

Interestingly, whilst the predicted survival for each HAP stage in this study was similar to that of previously published data [11], wider OS differences were observed in the poor prognostic group according to the ART score (22 and 29 months in our study; 6.6 and 8.1 months in the study by Sieghart et al. [12]). There are several reasons that may account for the survival differences observed. Firstly, our study includes a significantly higher proportion of BCLC-A in both datasets. The ART score was derived from dynamic changes in biochemical and radiological parameters following initial TACE, and patients with <2 TACE within 90 days and those achieving complete response after initial treatment were excluded in the original study. Equally, survival times were calculated from the day of the second TACE, and reassessment of liver function was carried out one day prior to second TACE [12]. The different purpose of our study, however, required a standardized comparison in homogenous populations unselected for clinico-pathologic features other than BCLC stage. This is a key point in the prognostic evaluation of the ART score, whose prognostic ability has been questioned in a previous analysis published by Kudo et al. which included patients from the same cohort included in this study [14]. The different selection criteria used for the two studies, however, are important in explaining the difference in our results. In the analysis by Kudo and in a subsequent, more comprehensive evaluation of the ART score in a cohort of 988 patients [25], only 12% of the patients underwent ≥2 TACE sessions within 90 days. In these patients the ART score failed to predict prognosis.

In our study, we obtained repeat blood tests and concurrent imaging reassessment 6-8 weeks after initial TACE, when patients presented for consideration of retreatment, a setting that – we believe – replicates a more appropriate time point for the clinical application of the ART score, rather than a pre-planned second TACE [12]. We also did not apply the 90-days interval criteria, considering the prognostic performance of the score in the whole patient population. Whilst the inherent differences in study design may account, in our opinion, for the heterogeneity in survival observed in ART score strata across studies, it should be emphasized that our study better approximates a routine clinical practice scenario where changes in the ART score could prompt changes in the management of unselected patients assessed following initial TACE. Whilst holding the undoubted advantage to be used sequentially to guide retreatment beyond initial TACE [10], based on our data, the ART score calculated after first TACE holds inferior accuracy in predicting short and long term prognosis compared to the HAP score.

However, given that both scores maintained a strong and independent prognostic value in our study, we were interested in testing their integrated effect in predicting OS in a large pooled cohort of 746 cases satisfying intermediate stage criteria. As shown in Supplementary Table 3, whilst the addition of the ART score is minimally or not significant at the extreme stages of the HAP prognostic algorithm, we surprisingly demonstrate the highest levels significance in the integration of the ART score in patients who belong to HAP stages B and C, suggesting that the dynamic changes in liver function and radiologic progression which compose the ART score may aid clinicians to reduce disease and treatment-related heterogeneity in survival outcomes, allowing to identify patients who are at higher risk of early mortality.

We propose an integrated algorithm with sequential use of both indices that may further refine prognostic prediction, with the HAP score being used as a screening tool to identify optimal candidates for initial TACE and the ART being sequentially used to identify early chemoembolization failure (Figure 3E). Whilst provocative, these conclusions should be further explored in adequately powered prospective studies.

As a secondary aim, we explored the prognostic in a separate analysis of a subgroup of patients with BCLC-C HCC, a patient subpopulation where TACE has been delivered with smaller but yet significant survival benefit in a number of retrospective case series [5]. Whilst the decision to treat was not supported by current BCLC guidelines, these patients received TACE at the discretion of the treating multidiscipilnary team, who felt, following case-by-case review, that TACE could have resulted in higher chances to achieve tumor control compared to other available therapies. We have shown that patients in the lower risk HAP stages, and those responding to initial TACE, achieve significant long-term benefit from treatment, albeit less than that in stage BCLC A/B. Interestingly, the ART score was not prognostic. Whilst the provision of TACE outside BCLC B stage should not be encouraged due to the lack of level I evidence, our findings are provocative in suggesting that a subset of patients with BCLC-C and HAP stage A+B, may benefit from TACE, a finding that may instigate further research.

The retrospective design and the relatively limited size of the training cohort stand as noteworthy limitations to our study. Perhaps unsurprisingly, the two cohorts demonstrated significant clinical heterogeneity, with Asian patients displaying lower tumor burden compared to Europeans, as a likely result of differing adherence to screening policies [14]. However, the process of independent validation in a large, independent cohort, the largest to have been utilized so far for the evaluation of prognostic models in intermediate-stage HCC, in conjunction with the levels of statistical significance achieved, confirms the validity, accuracy and generalizability of our results.

Additionally, we have analyzed objective and routinely collected data, which are likely to provide very low recall bias. The proportion of BCLC-A patients in both cohorts, who were offered TACE following multidisciplinary discussion confirming patient unsuitability for radical treatments, may account for the better survival figures observed here compared to other studies. Their inclusion is important in testing prognostic models in unselected TACE candidates. However, the significantly wider survival of this patient subgroup [26] warrants further validation studies focusing specifically on BCLC-A stage disease.

It is interesting to note that ROC curve analysis revealed AUROC values <0.75 in the prediction of landmark survival endpoints at 1 and 2 years, a finding that highlights the need to research into other factors other than those considered in the tested prognostic models as determinants of patients' survival after initial TACE. Similar considerations stem from the appraisal of the combined predictive ability of the combination of HAP and ART score, where Harrel's c index analysis produced an overall score of 0.69.

In summary, we have demonstrated the HAP score as an accurate predictor of short and long-term mortality, advocating a better-suited role in the initial screening of TACE candidates. We propose the use of the ART score, a more effective model for sequential risk-assessment, to assess suitability of patients prior to retreatment following initial TACE. We showed the potential for both scores to integrate in a uniform, objective and readily applicable selection strategy to optimize the provision of TACE in patients within intermediate-stage criteria, highlighting limitations within BCLC-A and C stage tumours.

Our study provides preliminary but stimulating evidence regarding the use of TACE in advanced disease, suggesting a potential benefit in a subgroup of patients with good HAP stage. Whilst thought provoking, our findings stem from a limited sample size, predominantly from a single-institution, and warrant verification in independent, adequately powered studies, especially given the role of the provision of further anticancer therapies post-TACE refractoriness in influencing patients' survival. Equally, the exclusion of patients with incomplete data might have lead to selection bias, an issue that further strengthens the need for prospective validation of our findings.

As the number of prognostic models is increasing rapidly based on retrospective evidence [27–30], further prospective clinical trials should be instigated to confirm the clinical utility of the proposed prognostic biomarkers in the management of intermediate stage HCC.

## PATIENTS AND METHODS

### Patient characteristics

Our training set population was retrospectively collected and consisted of a total of 83 subjects including 58 consecutive patients treated with TACE between 2004 and 2013 at the academic Liver Unit in Novara (Italy), and patients eligible for the assessment of the ART score (*n=25*) obtained from a larger database of 64 consecutive patients treated at the Hammersmith Hospital, Imperial College London (UK) between 2001 and 2012. Patients exceeding BCLC-B criteria, i.e. with segmental portal vein thrombosis (*n=4*) or Child C cirrhosis (*n=5*) were excluded from the primary analysis (Figure 2).

The diagnosis of HCC was made according to the American Association for the Study of the Liver criteria [16]. Patients demographics, full blood count, liver function tests, AFP, tumor staging, Child-Turcotte-Pugh (CTP) class, Barcelona Clinic Liver Cancer (BCLC) [1] and Cancer of the Liver Italian Program (CLIP) [17] scores were collected prior to treatment. All patients had a performance status of zero. OS was calculated from the time of the first TACE to the time of death or last clinical follow up. Calculation of the HAP score followed the criteria published by Kadalayil et al [11]. Calculation of the ART score followed previously published criteria [12]: both biochemical and radiologic parameters were obtained 6-8 weeks following initial TACE. Multiphase contrast-enhanced computed tomography (CT) images were reported by a senior radiologist blinded to survival data in accordance with modified Response Evaluation in Solid Tumors (mRECIST) criteria [18]. At both institutions TACE protocol was planned following multidisciplinary review of clinical data and staging CT and confirmed on pre-treatment hepatic arterial angiogram. Depending on tumour burden and vascular anatomy, TACE was administered selectively or super-selectively using a 2.7-2.8 Fr microcatheter, which served for intraarterial infusion of doxorubicin emulsified in lipiodol followed by embolization with gelatin sponge particles: in total. TACE was super-selective in a total of 49 patients (59%), selective in 28 (34%) whereas 6 patients (7%) had lobar TACE. Following TACE-refractoriness a total of 28 patients received sorafenib after TACE refractoriness (34%), whilst 6 had radiofrequency ablation of the residual disease (7%) and 2 had surgical resection (2%). All the remaining patients received best supportive care only.

For the purpose of this study we defined TACE refractoriness as a pattern of disease progression that would prevent safe and effective treatment with repeat TACE. This included the emergence of extrahepatic disease progression, multifocal intrahepatic disease progression no longer amenable to TACE or loss of adequate arterial vascular access feeding the residual viable disease.

### Validation of prognostic scores

The validation dataset was constructed as shown in Figure 2. This included a prospective series of 79 cases of HCC diagnosed at St. Mary's Hospital, Catholic University of Korea (Seoul, Republic of Korea) between June 2011 and July 2012 and a second retrospectively collected dataset of 644 consecutive patients with unresectable HCC treated with TACE at the Kinki University Faculty of Medicine (Osaka, Japan) between January 2004 and August 2013. In the Japanese subgroup chemoembolization was performed using 20-50 mg of epirubicin or 50-100 mg of cisplatin emulsified with lipiodol and gelatin sponge particles, for the Korean patients the TACE protocol consisted in the infusion of doxorubicin (50 mg) or combined epirubicin (50 mg) and cisplatin (60 mg) in a mixture of lipiodol followed by gelatin sponge embolization. Patients received intra-arterial treatment as appropriate for tumour burden, similarly to the training set. In total 572 patients (80%) had selective TACE, whilst the remaining 151 (20%) had lobar TACE. Response to treatment by mRECIST criteria was assessed 6-8 weeks after TACE, reported by a senior radiologist blinded to survival data.

Treatment data post-TACE refractoriness was collected. In the Korean sub-cohort, patients were accrued prior to the clinical availability of sorafenib and were treated with hepatic intra-arterial chemotherapy (HAIC) with epirubicin (50 mg) and cisplatin (60 mg) as previously described [19]. In the Japanese group, HAIC or systemic treatment with sorafenib or TS-1 were offered in patients displaying progressive disease no longer amenable to TACE. In total, 145 patients (20%) receive HAIC, whilst 148 (20%) were offered sorafenib and 33 (5%) received TS-1, whilst the remaining patients received best supportive care only. The study was approved by the local Research Ethics Committees and conducted in accordance to the principles of the Declaration of Helsinki. All institutions used a “treatment on demand” TACE schedule and no TACE was delivered in the presence of complete radiologic response.

### Statistical analysis

Kaplan-Meier statistics and Log-rank test were used to study the impact of the different clinical factors associated with OS on univariable analysis, with significant variables (*p<0.05*) being further tested on a multivariable stepwise backward Cox regression model. We used Harrell's rms packages to identify a subset of predictors by backward elimination [20], estimating the confidence intervals of the c-index statistics via bootstrapping (150 iterations). The proportional hazards assumption was tested by including the variable of interest and the product of the time varying variable constructed from the variable of interest in the Cox regression model. A resulting p>0.05 confirmed that the proportional hazards assumption over time was satisfied. The receiver operating characteristic (ROC) curve method was used to compare the discriminative ability of candidate variables in predicting 1 and 2-year mortality, with the area under the ROC curve (AUROC) being used to rank the prognostic models based on their predictive accuracy. Statistical analyses were performed using SPSS package version 11.5 5 (SPSS Inc., Chicago, IL, USA). R statistical package was used for c-index analysis [21].

## SUPPLEMENTARY FIGURES AND TABLES


