# Supplementary material for: Combined sequential use of HAP and ART scores to predict survival outcome and treatment failure following chemoembolization in hepatocellular carcinoma: a multi-center comparative study
Source: Oncotarget. 2016 May 26;7(28):44705–18. doi: 10.18632/oncotarget.9604 (PMC5190130; doi:10.18632/oncotarget.9604)
Supplement: Supplementary file 1 [file oncotarget-07-44705-s001.pdf]

SUPPLEMENTARY FIGURES AND TABLES

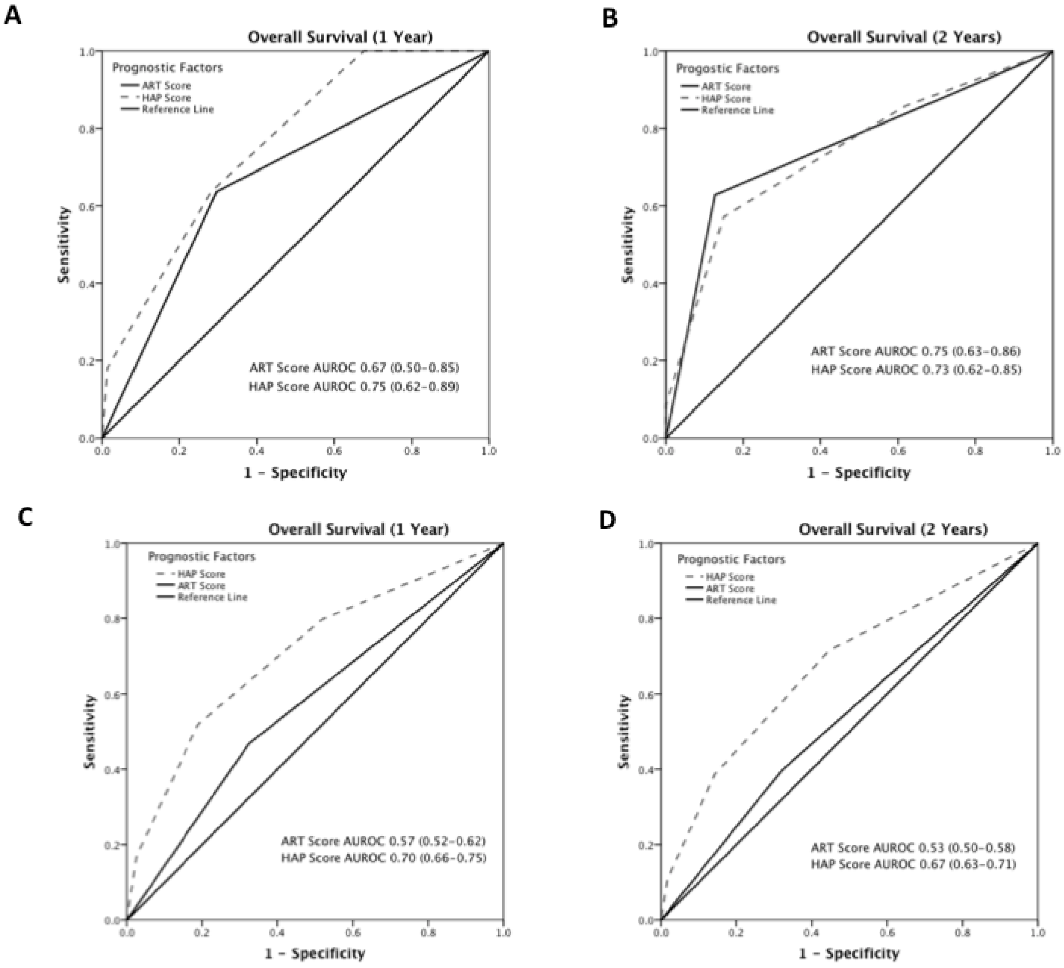

Supplementary Figure S1: Receiver operator characteristic (ROC) curves comparing the performance of HAP and ART score in predicting mortality at 1 and 2 years across the training (Panels A, B) and validation sets (Panels C, D).

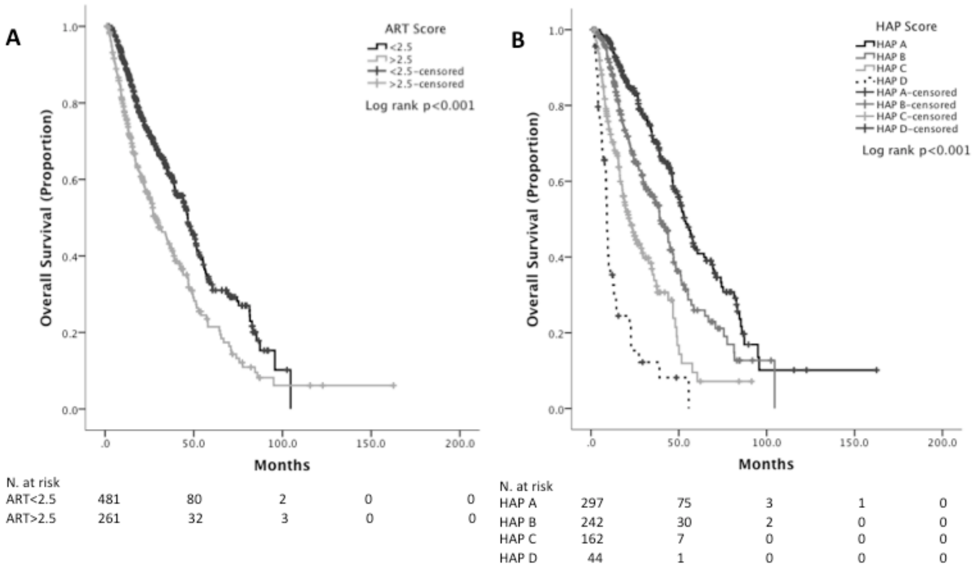

Supplementary Figure S2: Kaplan Meier curve analysis confirming the value of ART (Panel A) and HAP score (Panel B) as predictors of overall survival in HCC in the entire study population (n=746).

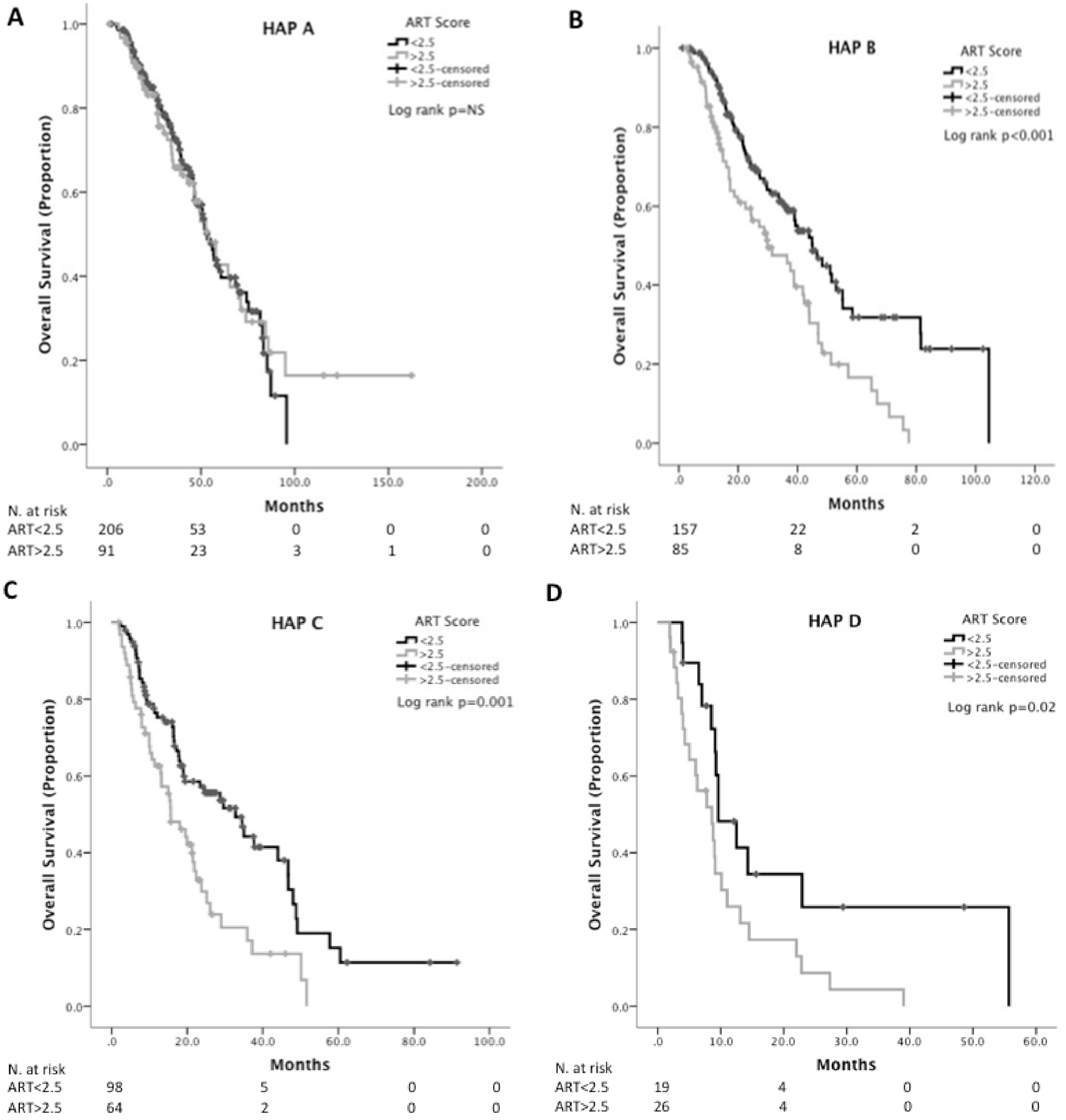

Supplementary Figure S3: Kaplan Meier curve analysis testing the prognostic value of the ART score stratified by HAP score.

Supplementary Table S1: Multivariate analysis of prognostic factors of overall survival (Training Set)

Variable		HR	95%CI	B	p-value
ART Score	<2.5	1.0			
	>2.5	2.9	1.3-6.9	1.0	0.01
HAP Score	A	1.0			
	B	3.2	1.0-9.8	1.1	0.03
	C	5.7	1.7-19.3	1.7	0.005
	D	76.2	10.7-544.4	4.3	<0.001

Supplementary Table S2: Multivariate analysis of prognostic factors of overall survival (Validation Set)

Variable		HR	95%CI	B	p-value
ART Score	<2.5	1.0			
	>2.5	1.5	1.3-1.9	1.0	<0.001
HAP Score	A	1.0			
	B	1.6	1.3-2.1	0.5	<0.001
	C	3.4	2.5-4.5	1.2	<0.001
	D	6.9	4.6-10.2	1.9	<0.001

Supplementary Table S3: The combined sequential use of HAP and ART scores refines the prognostic prediction of patients with intermediate stage HCC following initial TACE: pooled analysis of Training and Validation sets (n=746)

HAP Score	ART Score	N	Median OS (months)	95% CI	Log Rank (Mantel Cox Chi-square)	p value
**A**	<2.5	206	53.2	48.4-58.0	0.002	0.96
	>2.5	91	54.0	44.6-63.3		
**B**	<2.5	157	45.0	35.9-54.0	13.0	<0.001
	>2.5	85	30.0	17.6-42.1		
**C**	<2.5	98	32.8	22.0-43.5	11.4	0.001
	>2.5	64	15.7	10.4-20.8		
**D**	<2.5	19	9.6	5.5-13.7	5.19	0.02
	>2.5	26	8.6	4.7-12.4		
